# Efficacy and safety of physical therapy in patients with stage III COPD during ambulatory rehabilitation

**DOI:** 10.25122/jml-2023-0237

**Published:** 2023-12

**Authors:** Ulyana Kuz, Svitlana Maliuvanchuk, Roman Herych, Petro Herych

**Affiliations:** 1 Department of Traumatology, Orthopedics and Emergency War Surgery, Ivano-Frankivsk National Medical University, Ivano-Frankivsk, Ukraine; 2 Department of Pharmaceutical Management, Drug Technology and Pharmacognosy, Ivano-Frankivsk National Medical University, Ivano-Frankivsk, Ukraine; 3 Department of Internal Medicine №1, Clinical Immunology and Allergology, named after Academician Neyko EM, Ivano-Frankivsk National Medical University, Ivano-Frankivsk, Ukraine; 4 Department of Physical Therapy and Occupational Therapy, Vasyl Stefanyk Precarpathian National University, Ivano-Frankivsk, Ukraine

**Keywords:** chronic obstructive pulmonary disease, physical therapy, rehabilitation program, BBI-P - Pignet Body-Built Index, BMI - Body Mass Index, COPD - Chronic Obstructive Pulmonary Disease, FC - Functional Class, FEV - Forced Expiratory Volume, FEV1 - First Second of Forced Expiration, FEV1/FVC - First Second of Forced Expiration/Forced Vital Capacity, RDt - Ruffier-Dickson Test, Rt - Ruffier Test, VCI - Vital Capacity Index

## Abstract

This study aimed to determine the efficacy of a combined physical therapy and pharmacological treatment for patients recovering from stage III COPD exacerbation. The efficacy of the rehabilitation program was assessed using anthropological parameters, physical condition, respiratory system function, and functional endurance capacity. Data were collected from 39 patients with stage III COPD who underwent the rehabilitation program. Physical and anthropometric assessments were conducted using the Quetelet Body Mass Index, the Pignet Index, and the Vital Capacity Index (VCI). The functional capacities of the cardiorespiratory system were measured before and after the rehabilitation program using the Ruffier and Ruffier-Dickson tests and the hypoxic Shtange and Genchi tests. Exercise tolerance was evaluated using the Harvard Step Test and the Six-Minute Walk Distance Test (6MWD). Statistical analysis was conducted using the non-parametric Mann–Whitney U test for independent and dependent groups. Participants were randomly divided into two groups for rehabilitation: Group I received standard therapy and a routine physical therapy program, while Group II was given standard therapy along with a modified physical therapy regimen. There was a slight improvement in patient condition during the rehabilitation period for both groups. However, there was a low compliance rate for smoking cessation among the majority of patients, with some reducing their daily cigarette intake. Further long-time research is required to determine the efficacy of the proposed physical therapy program in combination with basic pharmacological therapy. The study suggests incorporating psychotherapeutic sessions and occupational therapy into future rehabilitation programs.

## INTRODUCTION

Chronic obstructive pulmonary disease (COPD) is a debilitating pathology adversely affecting patient quality of life and imposing a considerable socio-economic burden [1-3]. This condition, exacerbated by environmental factors such as pollution from motor vehicles and industrial sources, smoking, inadequate social and living conditions, as well as genetic predispositions to respiratory issues, has led to a rapid increase in COPD incidence. As of 2019, the global incidence of COPD among the population aged 30-79 was estimated at 10.3% [4], with rates in Ukraine slightly higher at 11.7% [5]. COPD is diagnosed in approximately 4-6% of the adult population worldwide, and its mortality rate has risen by 11.6% from 1990 to 2015 [6]. Addressing this growing concern requires comprehensive treatment and rehabilitation approaches aimed at reducing mortality and enhancing the quality of life for patients with COPD.

This study investigated the efficacy of a modified physical therapy program for patients with stage III COPD recovering from exacerbation.

## MATERIAL AND METHODS

### Research design and setting

This study was conducted across three outpatient facilities: Ivano-Frankivsk Regional Clinical Hospital, Ivano-Frankivsk Regional Phthisiopulmonology Center, and Ivano-Frankivsk City Polyclinic No.1. We included 39 patients with severe degree COPD who had completed a full inpatient treatment course for disease exacerbation and were continuing with outpatient basic therapy.

### Study framework

We grounded our research in a comprehensive review of relevant literature, analysis of previous patient examinations, and current treatment guidelines. To assess the effectiveness of the rehabilitation program, we conducted regular evaluations at specific intervals, starting from the initiation of the rehabilitation program (Day 1) to its conclusion (Day 14).

### Participant eligibility and recruitment

Patients were included based on a comprehensive review of their medical records to identify those with a history of acute coronary syndromes or prior myocardial infarction. The inclusion criteria were as follows: age over 40 years; COPD classified as grade III during the remission phase (FEV1 30–70% of the predicted value and FEV1/FVC < 70%); and an improvement in FEV1 of less than 12% (< 200 mL) after inhaling a short-acting β2-agonist compared to the baseline value. The exclusion criteria included COPD grades I, II, and IV, the presence of asthma, allergic rhinitis, pneumonia, acute coronary syndromes (unstable angina and myocardial infarction), coronary revascularization or cerebral stroke within the last 6 months, signs of heart failure classified as functional class III-IV, a left ventricle ejection fraction of less than 45%, clinically significant heart defects, persistent atrial fibrillation, impaired liver and kidney functions, concomitant stage III-IV hypertension, diabetes mellitus, and the inability to correctly perform a respiratory maneuver during the external respiration function test.

### Study design

Participants were blindly randomized into two groups using the Randomizer program: Group I (main, n=20) received standard therapy and a standard physical therapy program, and Group II (comparison, n=19) received standard therapy and a modified physical therapy program. Basic therapy was prescribed according to the local protocol [6]. In addition to the basic therapy of COPD, all patients received the new-generation medicine roflumilast based on its proven efficacy in our previous studies [7].

### Physical therapy management approaches

**Figure 1:**
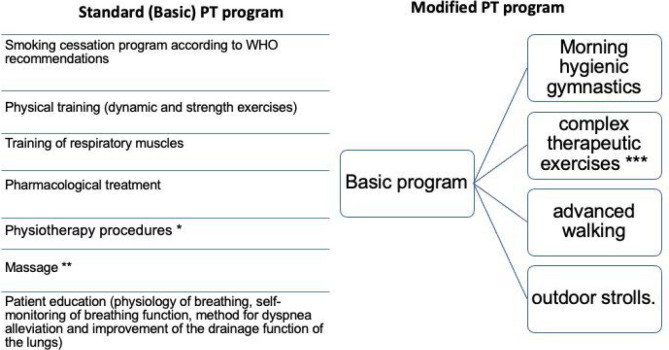
Rehabilitation scheme for patients included in the study * Application of ozokerite on the interscapular area, and aerosol and electro aerosol therapy were also used (1-2% solution of baking soda, sea salt, saline-alkaline mineral water, decoctions of plantain, coltsfoot, sage, mucolytic agents). The duration of the procedure was 15 to 30 minutes. **stroking, squeezing, rubbing, and kneading techniques were used. Additionally, vibration, hitting, and shaking techniques were used as well. *** Endurance and rhythmic exercise: The program incorporated exercises designed to gradually increase patient endurance. These activities were uniquely combined with choreographic elements to make the sessions engaging and enjoyable. Exercises varied in speed, rhythm, direction, and amplitude of movements, and were conducted to the accompaniment of rhythmic music styles such as foxtrot, Charleston, tango, disco, rock-n-roll, and breakdance.

All patients received basic physical therapy according to the World Health Organization (WHO) recommendations [8]. The comparison group, on the other hand, participated in a novel physical therapy program developed for this study (Figure 1).

Key components of the therapy included:

1. **Topical and inhalation treatments:** Patients received ozokerite applications to the interscapular region, combined with aerosol and electro-aerosol therapy. These therapies utilized a 1-2% solution of agents such as baking soda, sea salt, saline-alkaline mineral water, herbal decoctions from plantain, coltsfoot, sage, and mucolytic agents. Each session lasted between 15 to 30 minutes.
2. **Manual therapy techniques:** Various manual therapy techniques were employed, including gentle strokes, squeezes, rubs, and kneading. To complement these, more dynamic methods such as vibration, tapping, and shaking were also integrated into the treatment protocol.
3. **Endurance and rhythmic exercise:** The program incorporated exercises designed to gradually increase patient endurance. These activities were uniquely combined with choreographic elements to make the sessions engaging and enjoyable. Exercises varied in speed, rhythm, direction, and amplitude of movements and were conducted to the accompaniment of rhythmic music styles such as foxtrot, Charleston, tango, disco, rock-n-roll, and breakdance.

### Outcome evaluation

The anthropometric parameters were measured using standard techniques: body mass index (BMI) was calculated using the Quetelet formula [9], Pignet's body-built index [10], and vital capacity index [11]. Respiratory rate, measured as the number of breaths per minute, and the functional capabilities of the cardiorespiratory system were assessed using the Ruffier and Ruffier-Dickson tests [12], along with the Shtange test (breath-holding at inhalation) and the Genchi test (breath-holding at exhalation) [13]. To evaluate patients' exercise tolerance and the functional class (FC) of circulatory insufficiency, the six-minute walk test [14] and the Harvard step test were employed. Calculations were performed for the Ruffier and Ruffier-Dickson tests, the hypoxic Shtange and Genchi tests, and the mean values of the external respiration function parameters.

### Statistical analysis

Data were analyzed using Statistica 6.0 software. Normality was verified using the Shapiro–Wilk test and Kolmogorov–Smirnov test. The non-parametric Mann–Whitney U test was used for independent and dependent groups.

## RESULTS

**Figure 2:**
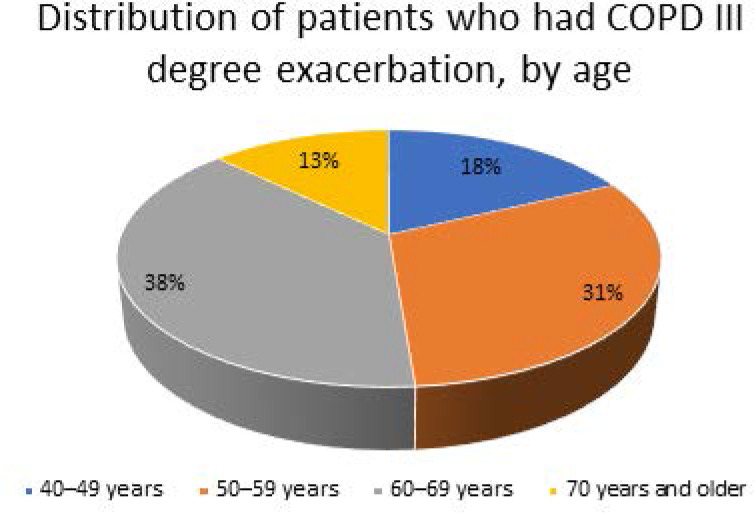
Distribution of study participants by age

All participants in the study were men, with an average age of 58.75±6.86 years. The comparison group was slightly older on average (59.2±8.1 years) compared to the main group (58.3±6.2 years). However, the differences were not statistically significant (P>0.05) (Figure 2).

Most patients were middle-aged individuals who wanted to remain employed and socially active. A significant proportion, 74.35% (29 patients), attributed their COPD exacerbation to acute respiratory viral infections. Exacerbations were also caused by other risk factors, including the common cold, non-compliance with treatment regimens, harmful habits, and hazardous working conditions (exposure to occupational risk factors). In 7 (17.94%) patients, the cause of COPD exacerbation could not be established. Furthermore, most patients with COPD exacerbation did not adhere to the prescribed basic therapy, primarily due to low compliance and the high cost of medications. The average rate of COPD exacerbations during the past year among the patients examined was 2.0±0.3 episodes, with the average duration of inpatient treatment being 14.5±1.7 days. In 16 patients (41.02%), the most recent COPD exacerbation was the third occurrence within the year, and in 5 patients (12.82%), it was the fourth time.

**Figure 3:**
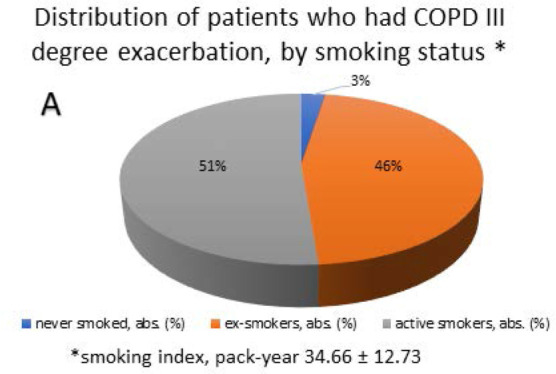
Distribution of study participants by smoking status

The progression of COPD degree is associated with a large number of factors, one of the most relevant being smoking status. Among the participants, there was only one patient (2.56%) who had never smoked, 18 (46.15%) patients were ex-smokers, and 20 (51.28%) patients were active smokers at the time of the study (Figure 3), with an average smoking history of 30.16±1.89 years and a smoking index of 34.66±12.73 pack-years.

**Figure 4:**
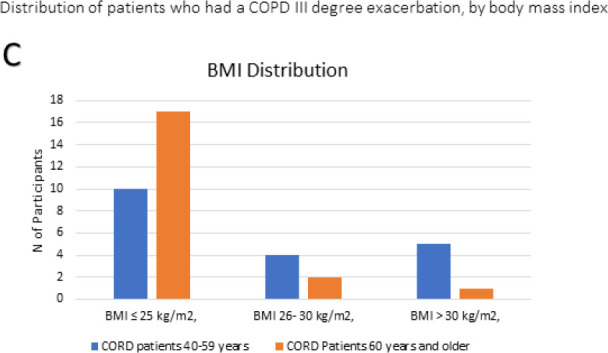
Corresponding BMI distribution between age groups: 40-59 years vs. ≥60 years

Also, it is well-known that one of the risk factors for systemic effects in COPD patients, in particular for the development of stable ischemic heart disease, is excessive body weight [15]. Additionally, the distribution of age and BMI highlighted a higher incidence of overweight and obesity among participants aged 40–59, whereas participants over 60 generally had lower body weights, aligning with the systemic weight loss observed in advanced COPD stages (Figure 4). At the end of the rehabilitation program, slight improvements were observed in patients from the comparison group (Table 1).

**Table 1:** Physiotherapy outcomes on anthropometric and respiratory indices before and after the intervention

Parameters	Study periods				
	Initial examination	End of examination	Initial examination		End of examination
	Main group		Comparison group		
BMI, units (U)	17.3±3.32	18.4±3.42	17.3±3.32	19.1±3.44	
BBI-P, units (U)	29.92±3.3	27.2±3.2	29.92±3.3	25.3±3.2	
VCI, mL/kg	49.47±6.10	52.60±6.86	49.47±6.10*	55.2±6.88*	

* – significant difference P<0.05 – P<0.001 compared to baseline data

In the main group, the BMI calculated using the Quetelet formula showed a slight increase in body weight by the end of the study. However, this increase was not statistically significant (P>0.05). A similar trend was observed in the comparison group, suggesting that participants were approaching a normal body weight. Although most participants did not reach the normal BMI range, they did manage to achieve a modest weight gain successfully.

BBI-P was improved by 9.1% in the main group, but it was not significant (P>0.1). Similarly, the comparison group improved body build strength by 18.26%, with average values of the body build index of 25.3±3.2 units. However, these changes were not statistically significant compared to baseline measurements (P>0.05).

In the main group, the VCI increased, but the difference compared to the baseline values was not statistically significant (P>0.05). Conversely, in the comparison group, there was a significant improvement in this parameter (P<0.05), indicating an improvement in lung tissue elasticity, reflected by an increase in vital capacity. This finding suggests that not only weight loss but also the improvement of lung tissue properties contributes to enhanced respiratory function.

To determine the functional state of the respiratory and cardiovascular systems in patients, the Shtange and Genchi functional tests and Ruffier and Ruffier-Dickson functional tests were performed, and the values obtained indicated an improvement in the functioning of the respiratory and cardiovascular systems (Table 2).

**Table 2:** Changes in respiratory and cardiovascular function following physical therapy among both groups

Parameters	Study periods			
	Initial examination	End of examination	Initial examination	End of examination
	Main group		Comparison group	
Ruffier index, conventional units (CU)	9.4±1.5	8.8±1.4	10.3±1.8*	8.2±1.2*
Ruffier-Dickson index, conventional units (CU)	7.4±1.2	6.8±1.02	7.8±1.3	5.9±0.89
Shtange test, seconds (sec)	36.8±9.6	40.1±9.8	36.8±9.6	41.4±10.2
Genchi test, seconds (sec)	14.4±1.9	17.2±2.1	14.4±1.9	21.4±2.1
Harvard step test, conventional units (CU)	56.8±9.8	64.3±10.3	56.8±9.8	67.5±12.3
6MWT, minutes (min)	268.4±34.6	316.4±36.8	268.4±34.6*	346.4±38.4*
Respiratory rate (number of breaths per minute)	20.5±3.52	19.8±3.48	21.3±3.83	19.6±3.52
First second of forced expiration (%)	49.5±7.27	58.9±7.21	49.4±7.25	58.5±7.32
First second of forced expiration/ forced vital capacity index	0.51±0.26	0.42±0.27	0.48±0.29	0.40±0.26

* – significant difference compared with the control group (P<0.05)

According to the study results, the Ruffier index in the main group at the end of the study was 8.8±1.4 CU, lower by 6.39% compared to the baseline data (9.4±1.5 CU). However, this difference was not statistically significant. The mean values of the Ruffier index were 8.2±1.4 CU in the comparison group, which was significantly lower by 20.39% compared to the baseline values and almost reached the values of the comparison group (7.8±1.5 CU).

The mean values of the Ruffier-Dickson index in the comparison group at the end of the study improved by 13.24% and were equal to 5.9±0.89 CU. Although the mean value was better compared to the parameters in the main group (6.8±1.02 CU), it was not statistically significant (t=0.66; P>0.1).

After analyzing the results of the modified physical therapy, an increase in cardiovascular system endurance was observed in both groups. However, there was a significant difference in the mean values of the cardiovascular system capacity only in the comparison group. These results imply that the physical therapy program proposed in this study may be only mildly effective compared to the standard one. In order to determine the functional state of the respiratory system after the therapeutic intervention, Shtange and Genchi tests were used. The resulting mean scores for both tests were categorized as "very weak" to "weak," suggesting a limited functional capacity of the respiratory and circulatory systems and a lower tolerance to hypoxia. In particular, the mean values of the Shtange test parameter in the main group were 40.1±9.8 sec and 41.4±10.2 sec in the comparison group, which is significantly less compared to normal ranges (52.3±5.4 sec).

The mean values of the Genchi test in the main group at the end of the study were 17.2±2.1 sec, and in the comparison group, 21.4±2.1 sec. These values, along with the values obtained in the Shtange test, indicate the insufficient functional state of the respiratory system compared to individuals in the control group but suggest an improvement in the functioning of the external respiration compared to baseline data.

The 6MWT, an effective measure of general physical endurance, showed that patients in the main group improved to 316.4±36.8 m, a 15.18% increase from baseline that was not statistically significant (P>0.05). In contrast, the performance in the comparison group significantly improved to 346.4±38.4 m (t=1.53; P<0.05), suggesting enhanced physical endurance. These findings underscore the potential of tailored physical therapy to enhance physical capacity in patients with COPD and support extending such therapeutic strategies to other settings, such as rehabilitation centers or home-based care.

## DISCUSSION

COPD is a debilitating disease that affects not only the lungs but also the entire body. It can cause bone and joint disorders such as sarcopenia and osteoporosis, leading to decreased mobility and quality of life [16]. COPD also increases the risk of cardiovascular complications, which can be life-threatening [17]. Moreover, it can lead to psychological problems such as depression and anxiety, further impacting the well-being of patients. It is important to raise awareness about the harmful effects and to take preventive measures to avoid such complications. Therefore, COPD is a disease that requires accurate, complex management [18].

The rapid advancements in clinical pharmacology and the development of new, highly effective treatment regimens have led to remarkable success in managing exacerbations. It is evident that a high smoking index and prolonged duration of smoking contribute to the negative effects of cigarette smoke components on the arterial walls, including coronary arteries. This results in an increase in systemic inflammation in the cardiovascular system, not just inside the respiratory system [19]. This understanding has underscored the inclusion of medications like Roflumilast in treatment protocols. Recent clinical guidelines emphasize the pharmacological approach together with non-pharmacological management to enhance the quality of life and reduce exacerbation frequency in patients with COPD. Our study aimed to establish a comprehensive rehabilitation strategy for COPD management. Despite efforts to educate patients on medication adherence, challenges persist, with a significant proportion of patients continuing to use medication irregularly or incompletely. Low adherence to treatment is an up-to-date challenge all around the world. Nearly half of patients with COPD have moderate and high compliance to long-term therapy and continuous exacerbation remission periods [20]. The remaining patients experience poor outcomes and reduced quality of life. Given this, it can be hypothesized that including an occupational therapy specialist in the rehabilitation program could be beneficial. Such a specialist would design individualized approaches to enhance pharmacological adherence in home settings, a strategy that has been supported by findings from other researchers [21, 22].

In addition to pharmacological treatment adherence, smoking cessation represents a critical component of non-pharmacological intervention that requires complex intervention. Our efforts and those of our patients to achieve complete smoking cessation faced significant challenges, resulting in less than satisfactory outcomes. The literature review suggests there are limited options for overcoming this addiction. Certain medications can reduce smoking frequency, but they often contain nicotine, which is associated with adverse cardiovascular effects [23]. Among non-pharmacological strategies, the efficacy of some interventions remains under debate. However, cognitive behavioral therapy and smoking cessation websites have shown promise as effective interventions [24]. Our study faced limitations in incorporating occupational therapists and psychologists into the rehabilitation process, as these services are not covered by state medical insurance, highlighting a significant gap in comprehensive care for individuals seeking to quit smoking.

The chronic progression of the disease often leads to exacerbated dyspnea and a consequent reduction in muscle mass and strength. This deterioration can trigger significant social disengagement and a decline in quality of life. In our study, implementing a physical therapy program has demonstrated effectiveness in enhancing the functional endurance of patients with COPD, aligning with findings from previous research [25, 26]. Even within the limited timeframe of our study, participants in the comparison group experienced improvements in lung vital capacity. However, the study did not show a significant increase in BMI and BBI-P indexes, indicators of muscle development. BMI remains one of the main predictors of respiratory function decline in COPD, also known as the “obesity paradox” [27]. We hypothesize that further permanent physical training should help patients with COPD increase BMI.

During our study, we encountered some limitations that need to be addressed. Firstly, the sample size of potential participants was limited due to certain exclusion criteria, such as cardio-respiratory complications of COPD (such as asthma) or inner organ concomitant diseases. This led to a lack of diversity in the included population, which was not originally intended. Additionally, the follow-up observation period was shortened, which affected our ability to properly evaluate the efficacy and safety of a modified rehabilitation program. Going forward, we plan to redesign our physical therapy program to include occupational therapy intervention and encourage patients to undergo psychotherapy sessions for a more comprehensive approach. The scope and diversity of participants were limited by specific recruitment criteria, resulting in a cohort of only 39 male patients who fulfilled the inclusion requirements without meeting any exclusion criteria. No female patients were included. Furthermore, not all eligible patients were able to sign the informed consent. The study duration was intentionally shortened to facilitate a timely evaluation of the modified rehabilitation efficacy and safety, aiming to make prompt adjustments as needed.

## Conclusion

The comprehensive physical therapy program we developed significantly enhanced functional endurance in individuals with stage III COPD. Therefore, it could be implemented in clinical practice as it has shown high efficacy, safety, and feasibility and could be recommended for wide use in outpatient facilities and at home.

On the other hand, most patients had low compliance with smoking cessation, although some patients reduced the number of cigarettes per day. This observation underscores the necessity for continued research, particularly long-term studies, to verify the long-term efficacy of our physical therapy program when combined with standard pharmacological treatments. Moreover, integrating psychotherapeutic support and occupational therapy into the rehabilitation regimen could potentially enhance overall outcomes, providing a more holistic approach to COPD management and patient care.

Further longitudinal research is required to establish the efficacy of the physical therapy program in combination with basic pharmacological therapy. Incorporating psychotherapy sessions and occupational therapy into the rehabilitation program may offer a more comprehensive approach to managing COPD.
